# The Impact of U.S. Free Trade Agreements on Calorie Availability and Obesity: A Natural Experiment in Canada

**DOI:** 10.1016/j.amepre.2018.02.010

**Published:** 2018-05

**Authors:** Pepita Barlow, Martin McKee, David Stuckler

**Affiliations:** 1 Department of Sociology, University of Oxford, Oxford, United Kingdom; 2 Department of Health Services Research and Policy, London School of Hygiene and Tropical Medicine, London, United Kingdom; 3 Department of Policy Analysis and Public Management, Bocconi University, Milan, Italy

## Abstract

**Introduction:**

Globalization via free trade and investment agreements is often implicated in the obesity pandemic. Concerns center on how free trade and investment agreements increase population exposure to unhealthy, high-calorie diets, but existing studies preclude causal conclusions. Few studies of free trade and investment agreements and diets isolated their impact from confounding changes, and none examined any effect on caloric intake, despite its critical role in the etiology of obesity. This study addresses these limitations by analyzing a unique natural experiment arising from the exceptional circumstances surrounding the implementation of the 1989 Canada–U.S. Free Trade Agreement.

**Methods:**

Data from the UN (2017) were analyzed using fixed-effects regression models and the synthetic control method to estimate the impact of the Canada–U.S. Free Trade Agreement on calorie availability in Canada, 1978–2006, and coinciding increases in U.S. exports and investment in Canada’s food and beverage sector. The impact of changes to calorie availability on body weights was then modeled.

**Results:**

Calorie availability increased by ≅170 kilocalories per capita per day in Canada after the Canada–U.S. Free Trade Agreement. There was a coinciding rise in U.S. trade and investment in the Canadian food and beverage sector. This rise in calorie availability is estimated to account for an average weight gain of between 1.8 kg and 12.2 kg in the Canadian population, depending on sex and physical activity levels.

**Conclusions:**

The Canada–U.S. Free Trade Agreement was associated with a substantial rise in calorie availability in Canada. U.S. free trade and investment agreements can contribute to rising obesity and related diseases by pushing up caloric intake.

## Introduction

The escalating global prevalence of overweight and obesity, or “globesity,” is often described as a pandemic.^1^ Worldwide, it is estimated that rates of overweight and obesity combined rose by 27.5% for adults and 47.1% for children between 1980 and 2013.^2^ Globalization via free trade agreements (FTAs) is often implicated in this pandemic because of its role in spreading high-calorie diets rich in salt, sugar, and fat.^3^ These concerns have become increasingly prominent in recent years, as new FTAs have been negotiated at an unprecedented rate, rising from 22 active FTAs in 1990 to more than 270 in 2016.^4^ They include the Transatlantic Trade and Investment Partnership, a potential agreement between the U.S. and the European Union, and a possible United Kingdom–U.S. deal.^5^ Public health specialists have argued that new FTAs could worsen diets and exacerbate rising rates of obesity.^3^

However, a recent systematic review showed that evidence of a link between FTAs and unhealthy diets and obesity was methodologically and substantively limited.^6^ Methodologically, previous studies have not addressed critical challenges to causal inference when analyzing the impact of FTAs. One challenge is that FTAs are often implemented in response to major macroeconomic crises or alongside market-oriented policies, such as deregulation.^7^ These transformations can also influence diets, making it difficult to isolate the impact of FTAs.^8^ In addition, there is often a delay of several years between when an FTA is agreed upon and when it is implemented, making it difficult to identify the appropriate pre- and post-FTA cut off.^9^ Previous studies were unable to disentangle this complexity.

Substantively, previous analyses of FTAs and diets focused on a narrow range of outcomes: high-fructose corn syrup supply and sugar-sweetened beverage sales.^9^, ^10^, ^11^, ^12^ However, whether or not FTAs contribute to rising obesity depends, in part, on whether they increase peoples’ net caloric intake (i.e., caloric intake less caloric expenditure), as this plays a critical role in the etiology of obesity.^13^ FTAs may do so by facilitating trade in the food and beverage sector as they reduce trade barriers, such as tariffs (a type of trade tax) and non-tariff barriers, such as differences in technical or quality standards. FTAs can also boost domestic food and beverage production when barriers (such as a lack of investor protection) to foreign investment are removed.^3^, ^14^ These changes can, in turn, lead to lower prices; greater availability; and greater marketing of food, beverages, and their ingredients. These three factors can alter diets, as they affect the composition and quantity of food and beverage production and consumption.^3^

Whether or not these changes encourage higher caloric intake is likely to vary according to the partner country, and U.S. FTAs are especially likely to encourage elevated caloric intake because of the highly competitive processed food and caloric beverage industry in the U.S.^15^ Processed food and caloric beverages play an important role in increasing caloric intake, as they are often calorie dense, leading people to unknowingly consume too many calories, and highly palatable, encouraging further consumption. In addition, drinking caloric beverages can contribute to increased caloric intake, as it is rarely compensated for by an equivalent reduction in food consumption.^16^, ^17^, ^18^

This study addresses these gaps by analyzing a unique natural experiment, the Canada–U.S. Free Trade Agreement (CUSFTA) in 1989. This study tests the hypotheses that CUSFTA increased caloric intake in Canada and that these changes corresponded with increased U.S. exports and investment in the Canadian food and beverage sector.

Dunning^19^ identifies three criteria that characterize a natural experiment. First, exposure to the intervention (here the FTA) and control must be as-if random. In this way, it simulates a randomized trial, although assignment of the intervention is outside the researchers’ control. Second, the statistical models must be credible so that differences between intervention and control groups are not attributable to confounders, and third, the case must have substantive relevance.^19^ The following section describes how CUSFTA meets these criteria.

On January 1, 1989, CUSFTA came into force. CUSFTA reduced barriers to trade and investment between the U.S. and Canada in most sectors of the economy, including the food and beverage industry, as summarized in Appendix 1 (available online). CUSFTA was subsumed by the North American Free Trade Agreement on January 1, 1994, which changed few trade arrangements between the U.S. and Canada, as these were covered by CUSFTA.

CUSFTA is in many ways a unique natural experiment. First, CUSFTA is substantively relevant, as it was a blueprint for later FTAs.^20^ Second, CUSFTA was not part of a larger package of reforms or implemented in response to a macroeconomic crisis so, unlike most FTAs, it is not confounded by these changes.^7^ Third, the pre- and post-FTA periods are clearly demarcated, and fourth, CUSFTA was unanticipated. This is because the fate of CUSFTA was decided by the Canadian general election in 1988. This so-called Free Trade Election was very closely contested and centered on whether to implement CUSFTA.^21^ One side was pro-CUSFTA and the other against. No one could be certain who would win—and so whether CUSFTA would be implemented—until the outcome of the election in November 1988. This created a distinct pre- and post-FTA cut off and addresses issues created by potential anticipatory effects.

Fifth, CUSFTA’s implementation was as-if random. CUSFTA’s implementation was contingent on the outcome of the 1988 election. But the victory of the pro-CUSFTA party was a quasi-random event: most Canadians voted for parties that opposed CUSFTA, but the pro-CUSFTA party secured a marginal victory and implemented the FTA, as they won a majority of votes in two provinces that, because of Canada’s electoral formula, elected more seats than the remaining eight Canadian provinces combined.^22^ In addition, CUSFTA was implemented almost immediately after the election on January 1, 1989. Thus, CUSFTA was not implemented in response to any changes that occurred in Canada after the marginal victory.

This study evaluates the impact of CUSFTA on calorie availability in Canada, assesses whether changes in trade and investment potentially mediated this association, and simulates the impact of dietary changes on body weight.

## Methods

### Study Sample

Appendix 2 (available online) summarizes the data sources and variables used in the analysis. The impact of CUSFTA on Canadian diets was estimated using public and de-identified annual calorie availability data from the UN Food and Agricultural Office Statistics Office.^23^ This captures the total quantity of food and beverages available for human consumption in kilocalories (kcal) per capita per day. Calorie availability is a widely used proxy for consumption that is more widely available than individual survey measures, which were not available in Canada or on a cross-national basis during the study period.^24^ Data on country-level covariates of calorie availability were sourced from the World Bank World Development Indicators, 2015 Edition.^25^ Trade and investment data were from the U.S. Bureau of Economic Analysis and the U.S. Department of Agriculture.^26^, ^27^ Body weight and height data used for weight change modeling were based on data from the Canadian Health Promotion Survey in 1990 when data were first available.^28^

### Measures

A fixed-effects regression model was used to compare changes to calorie availability in Canada with comparison countries, 1978–2006. The model is given by:

$$Y_{\mathrm{it}}=\alpha_{0}+\gamma_{i}+{\beta D}_{\mathrm{it}}+{\theta X}_{\mathrm{it}}+\in_{\mathrm{it}}$$

where $Y_{\mathrm{it}}$ is calorie availability in country *i* at time *t*; $\alpha_{0}$ is the intercept; $\gamma_{i}$ is the country-specific fixed effect capturing unobserved, time-invariant factors that vary between countries and may impact diets. β is the coefficient of interest capturing the impact of CUSFTA. It is estimated using a dummy variable for the treatment status, where D=1 in Canada during the post-CUSFTA period 1989–2006 and D=0 otherwise. $X_{\mathrm{it}}$ is a vector of covariates with coefficients in the vector θ; following previous studies the models control for linear time trends, Gross Domestic Product per capita, and urbanization rates.^9^, ^10^, ^11^

**Figure 1:**
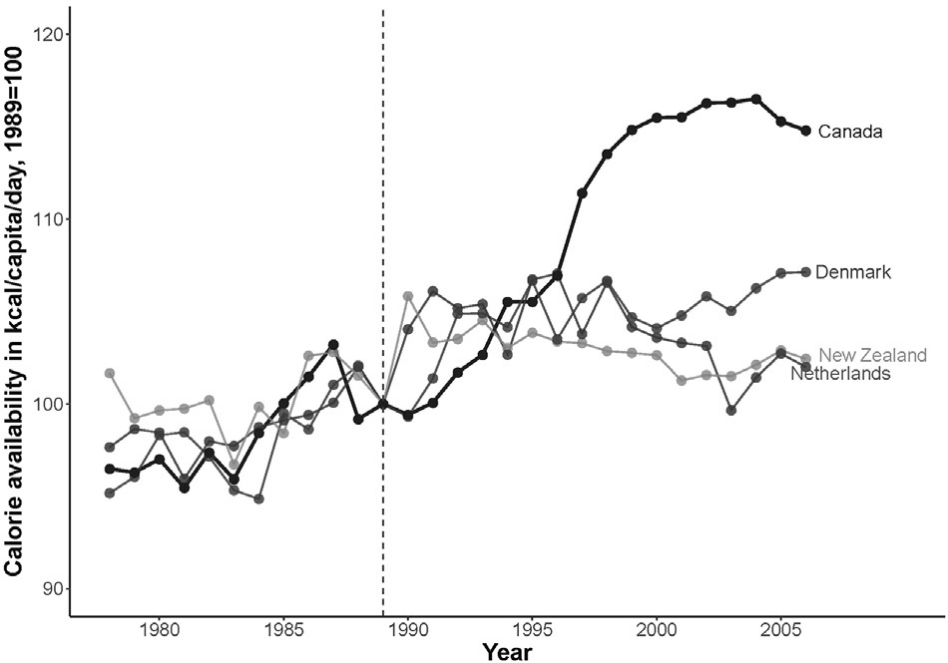
Normalized trends in calorie availability in Canada and comparison countries, 1978–2006. *Note:* Data from the Food and Agriculture Organization of the United Nations (2016).

A valid comparison country or countries should match on key parameters, not have received the U.S. FTA “treatment,” have available data, and exhibit parallel trends in the outcome variable in the period preceding the treatment.^19^, ^29^ Appendix 3 (available online) describes how these criteria were applied. Briefly, the sample of potential comparison countries was restricted to countries with available data that, like Canada, had high-income levels; were members of the World Trade Organization and Organization for Economic Co-operation and Development; and exhibited parallel trends in calorie availability before CUSFTA, but did not enter a U.S. FTA during the study period: Denmark, The Netherlands, and New Zealand. Figure 1 shows that the comparison countries had trends similar to Canada before CUSFTA. As a robustness test for the sensitivity of the results to the fixed-effects model’s identifying assumptions, the analysis was also conducted with a larger sample of countries and using the synthetic control method.^30^

### Statistical Analysis

The analysis was conducted in the period 1978–2006, beginning when calorie availability and covariate data were available for all countries in the sample and ending before the onset of the financial crisis in 2007–2008, as this created widespread macroeconomic and dietary changes.^31^

Potential mechanisms linking CUSFTA to changes in calorie availability were evaluated by analyzing trends in U.S. investment and trade with the Canadian food and beverage sector. Next, the impact of the estimated change in calorie availability attributable to CUSFTA on individuals’ body weight was simulated using widely applied models developed by Hall and Jordan.^32^ Weight gain was estimated assuming 100% and 50% pass-through from calorie availability to intake; Appendix 4 (available online) provides full details. Finally, additional sensitivity analyses tested the robustness of the results.

## Results

Figure 1 plots normalized trends in calorie availability in Canada and comparison countries and shows that the availability of calories increased markedly in Canada after CUSFTA. In Canada, calorie availability rose from 3,028.5 kcal/capita/day in 1988 just before CUSFTA was implemented to 3,491.0 kcal/capita/day in 2006. Thus, calorie availability was on average 343.1 (95% CI=294.3, 391.9) kcal/capita/day higher in Canada after CUSFTA compared with before CUSFTA. Since 1994, the rise in calorie availability in Canada far exceeded other countries, where calorie availability was on average 150.8 (95% CI=114.4, 187.1) kcal/capita/day higher post-CUSFTA following a period of weak economic performance in the late 1980s and early 1990s.^33^

Table 1 summarizes the results from the fixed-effects regression models and shows that CUSFTA was associated with a 170.3 (95% CI=73.0, 267.5) kcal/capita/day increase in calorie availability in Canada after adjusting for covariates.

**Table 1:** Estimated Effect of CUSFTA on Calorie Availability in Canada: Fixed-Effects Regression Results

**Variable**	**Model 1**	**Model 2**	**Model 3**
CUSFTA, coefficient (95% CI)	**343.1 (294.3, 391.9)**	**194.0 (119.2, 268.7)**	**170.3 (73.0, 267.5)**
US$100 increase in GDP per capita, coefficient (95% CI)	—	—	1.1 (–0.03, 2.3)
1% increase in rate of urbanization, coefficient (95% CI)	—	—	17.1 (–6.5, 40.8)
Controls for fixed effects?	Yes	Yes	Yes
Controls for time trends?	No	Yes	Yes
Country years	116	116	116
Adjusted *R*^2^	0.31	0.62	0.62

*Note:* Boldface indicates statistical significance (*p*<0.05). Models were estimated using cluster-robust SEs grouped at the country level. Results from the Hausman test firmly rejected the null hypothesis of independence between the random-effects estimate and the error term, favoring the fixed-effects over the random-effects estimator.

CUSFTA, Canada U.S. Free Trade Agreement; GDP, Gross Domestic Product.

U.S. investment in the Canadian food and beverage sector was on average US$1.82 billion (95% CI=US$1.18, US$2.46 billion) higher in the period 1989–2006 after CUSFTA compared with before CUSFTA (Appendix 5, available online). Calorie availability in Canada began rising 5 years after an increase in U.S. Foreign Direct Investment that started immediately after CUSFTA and stopped rising 5 years after the rise Foreign Direct Investment stopped in 1999 (Appendix 6, available online). Food and beverage trade between Canada and the U.S. also increased after CUSFTA, in both directions (Appendix 7, available online). U.S. food and beverage exports to Canada were US$5.26 billion (95% CI=US$4.89, US$5.62 billion) higher after CUSFTA.

Table 2 shows the results from the weight-gain modeling. CUSFTA was estimated to lead to a steady-state weight gain of between 1.8 kg and 9.3 kg for men and 2.0 kg and 12.2 kg for women aged 40 years depending on physical activity levels and assumed pass-through from calorie availability to intake.

**Table 2:** Estimated Increase in Body Weight by Sex and Physical Activity Level From a 170-kcal and 85-kcal Rise in Daily Caloric Intake

**Physical activity level**	**Sex**	**Estimated weight gain, kg**	
		**170 kcal/capita/day**	**85 kcal/capita/day**
Low^a^	Female	12.2	4.4
High^b^	Female	5.3	2.0
Low^a^	Male	9.3	3.9
High^b^	Male	4.0	1.8

*Note:* Figures show the estimated increase in body weight among males aged 40 years and figures once body weight reaches a steady state (i.e., after accounting for the dynamic physiological adaptations that occur during weight gain). Weight gain figures are based on average weight and height, by sex, adults aged 40 years in the Canadian Health Promotion Survey, 1990.^28^Appendix 4 (available online) provides full details.

a Equivalent to walking 2.2 miles per day at 3–4 miles per hour (mph).

b Equivalent to walking 17 miles per day at 3–4 mph. kcal, kilocalorie.

**Figure 2:**
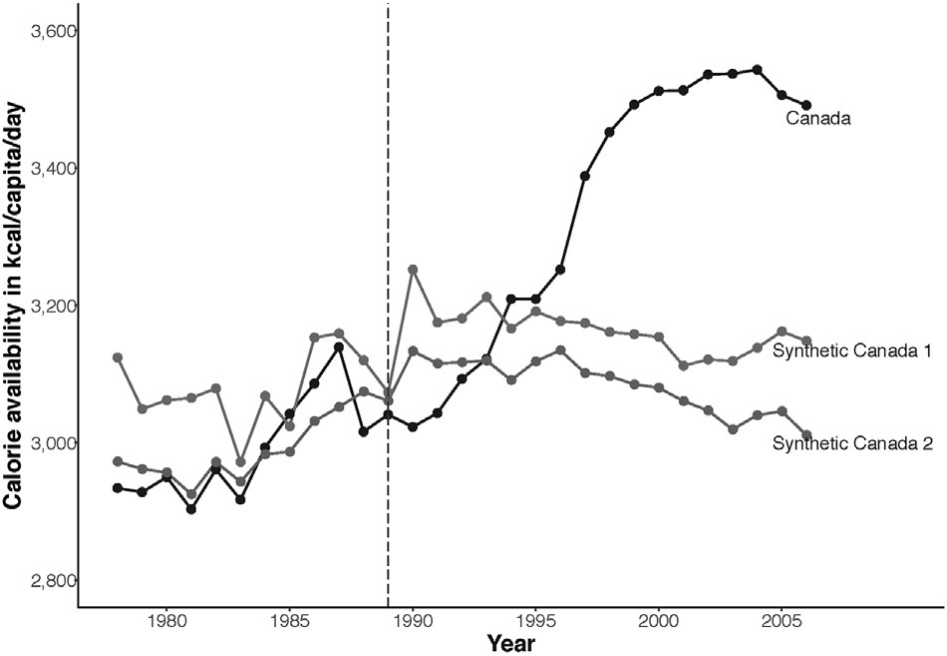
Synthetic control results. *Note:* Synthetic control 1 shows results using original sample of comparison countries. Synthetic control 2 shows results using a larger sample of Organisation for Economic Co-operation and Development comparison countries as the synthetic control method relaxes the parallel trends assumption. Appendix 7 (available online) provides full details.

Additional analyses tested the robustness of these results. First, as inferences from fixed-effects regression models may be sensitive to the sample selection criteria, the effect of CUSFTA was re-estimated after incorporating additional Organization for Economic Co-operation and Development countries in the sample, including the U.S. Results were consistent with the main analysis (Appendix 8, available online). Second, fixed-effect models implicitly assume that the differences between Canada and comparison countries can be captured by covariates included in the regression model, whereas Canada and comparison countries could differ in other ways, which might, at least partially, account for the results.^34^ This was addressed by re-estimating the impact of CUSFTA using an alternative model: the synthetic control method.^30^ Figure 2 and Appendix 9 (available online) show that the results were consistent with the main analysis, while reducing differences in characteristics between Canada and the counterfactual.

Second, an in-time placebo analysis was performed to test whether the results could be attributed to unobserved factors driving periodic changes in calorie availability. The coding of the CUSFTA variable was re-assigned to 1981 and then the models were re-estimated in the 1978–1988 period. The placebo CUSFTA had no perceivable impact on calorie availability (Appendix 10, available online). Third, although all countries experienced a decline in calorie availability just before CUSFTA, the results could be attributable to a recovery from to a pre-intervention dip in Canada. As recommended in Bertrand et al.,^35^ the models were re-estimated after excluding observations 2 years to either side of CUSFTA. The effect estimate for CUSFTA was consistent with the main analysis (Appendix 10, available online). Fourth, the sensitivity of the results to the inclusion of any specific country in the sample was evaluated by iteratively omitting each country from the sample and re-estimating the fixed-effects model. The effect estimate for CUSFTA was substantively comparable across sample specifications (Appendix 11, available online).

## Discussion

This analysis suggests that calorie availability in Canada increased by approximately 170 kcal/capita/day after CUSFTA. This coincided with a US$1.82 billion (95% CI=US$1.18, US$2.46 billion) increase in U.S. investment in the Canadian food and beverage industry and a US$5.26 billion (95% CI=US$4.89, US$5.62 billion) rise in food and beverage imports from the U.S. The rise in caloric availability was estimated to lead to an average weight gain of 1.8–9.3 kg for men and 2.0–12.2 kg for women who were aged 40 years, depending on their physical activity levels and assumed pass-through from availability to intake. These results were robust across different model and sample specifications.

This study suggests that CUSFTA altered dietary behavior substantially by increasing calorie availability in Canada. These findings are consistent with previous, narrower studies finding that trade agreements with the U.S. create food environments that more closely resemble the unhealthy obesogenic environment that pertains in the U.S.^6^

This analysis also advances previous research in three important ways. First, the study finds more robust evidence to suggest a causal impact of FTAs on diets. Unlike previous analyses, the exceptional circumstances surrounding CUSFTA’s implementation provided a unique quasi-experimental setting that created as-if random implementation, an isolated FTA, a clear pre- and post-intervention cut off, and overcame anticipatory effects.

Second, this analysis suggests that U.S. FTAs can impact the number of calories that are available and likely consumed. The estimated impact on weight gain is consistent with observed increases in obesity rates among Canadian adults, rising from 5.6% in 1985 to 14.7% in 2003.^36^ Of course, the rise in calorie availability after CUSFTA would not necessarily have contributed to rising obesity had calorie supplies been insufficient before CUSFTA, or had physical activity increased in parallel. Yet, calorie supplies met energy needs in 1984 before CUSFTA came into force.^37^ Furthermore, Bleich and colleagues^38^ reported that 100% of the rise in obesity in Canada from 1990 to 2002 was attributable to a 513 kcal/capita/day rise in calorie availability over the period, as there was no coinciding decline in physical activity. CUSFTA was associated with an approximately 170 kcal rise in calorie availability, which constitutes approximately 33% of the total increase, from 1990 to 2002. CUSFTA may have contributed up to 33% of the rise obesity during the period by pushing up caloric intake.

Finally, existing studies of FTAs and related liberalization policies have emphasized the role of trade rather than investment in mediating their consequences.^6^ Changes to calorie availability in Canada diverged from comparison countries since 1994 and so corresponded to changes in U.S. investment in the Canadian food and beverage sector at a 5-year time delay. This suggests that investment was at least as important as trade in mediating the impact of CUSFTA on diets, and that the time needed for increased investment to translate into increased production accounted for the delayed rise in calorie availability after CUSFTA.^39^

### Limitations

This study has several limitations. First, calorie availability is an imperfect measure of caloric intake and potential weight gain because of difficulties in estimating wastage and home production. Nevertheless, calorie availability is a widely used proxy for consumption that has several strengths compared with survey-based measurements that are subject to recall or social approval biases.^40^, ^41^ Second, it is not feasible to conduct a fully randomized experiment to assess the effects of FTAs. One or more factors that were beyond the researchers’ knowledge and control may account for the results. However, in situations where experimental manipulation is unfeasible, as is the case with FTAs, the natural experiment design used in this study is recommended as the best means for evaluating causal effects.^42^ Third, the models adjust for Gross Domestic Product per capita and urbanization, which were plausibly impacted by CUSFTA. The results were nevertheless consistent across model specifications with and without controls. The attenuation of the CUSFTA effect estimate when these variables were incorporated also suggests that they led to conservative estimates.

Canadian idiosyncrasies and contextual factors at the time CUSFTA was implemented may nevertheless limit the external validity of this analysis. However, this study may be informative, as CUSFTA was a blueprint for later FTAs.^20^ In addition, limited data availability precluded a direct analysis of changes to weight gain, related health outcomes, and their socioeconomic stratification. Future research should address these limitations.

## Conclusions

Notwithstanding its limitations, this study has important implications for policy. Public health scholars have long argued that dietary choices and obesity are influenced by food environments, which are, in turn, shaped by macrostructural factors.^43^, ^44^ This analysis suggests that FTAs can lead to a substantial rise in calorie availability and likely intake, which plays a critical role in the development of obesity. Thus, this study shows empirically how trade policy is a macrostructural driver of dietary behaviors.^44^ This paper also strengthens the legitimacy of growing concerns raised during FTA negotiations about the potentially detrimental impacts of U.S. FTAs and the need for greater coherence between nutrition and trade policy making.^5^, ^45^
