# Supplementary material for: The Impact of U.S. Free Trade Agreements on Calorie Availability and Obesity: A Natural Experiment in Canada
Source: Am J Prev Med. 2018 May;54(5):637–43. doi: 10.1016/j.amepre.2018.02.010 (PMC5906641; doi:10.1016/j.amepre.2018.02.010)
Supplement: Supplementary file 1 — Supplementary material [file mmc1.pdf]

Appendix 1. Summary of Trade and Investment Policy Changes in Canada Mandated by CUSFTA Effective From January 1, 1989

Policy	Policy change
Tariffs	Complete elimination of tariffs on U.S. imports over 10 years, except for tariffs on imports from third parties
Quotas and restrictions	Prohibition of restrictions and quotas on export quantities, export taxes, and subsidies
Standards and regulations	Standardized product and environmental regulations
National treatment	U.S. businesses in Canada are subject to the same regulatory conditions as domestic firms, including social responsibility regulations, taxation, and admission and establishment procedures
Investment protection	Rules that protect foreign investors from ‘expropriation’: a government policy that de facto substantially reduces a firm’s profits, unless the measure is designed to serve the public interest, is not discriminatory, or the investor is compensated for the losses suffered
Dispute settlement	Commonly agreed procedures for settling disputes over investment expropriation

Source: Text of the Canada-U.S. Free Trade Agreement (CUSFTA).[1]

Appendix 2. Summary of Variable Definitions and Data Sources

Variable	Measure	Source
Calorie availability	Kilocalories per capita per day	UN Food and Agricultural Organisation Statistics Office (FAOSTAT)
GDP per capita	Gross Domestic Product (GDP) per capita, measured in constant 2005 U.S. dollars and adjusted for differences purchasing power	World Bank World Development Indicators (2015 edition)
Urbanization	Proportion of the total population living in urban dwellings as a percentage of the overall population	World Bank World Development Indicators (2015 edition)
U.S.-Canada food and beverage trade	U.S. food exports to Canada and Canadian food exports to the U.S. measured at current prices in millions of U.S. dollars	U.S. Department of Agriculture (USDA)
U.S. investment in the Canadian food industry	U.S. investment position in the food and beverage industry in Canada on a historical-cost basis in millions of U.S. dollars	U.S. Bureau of Economic Analysis (BEA)
Weight, by sex, among men and women aged 40 years	Mean self-reported weight, in kg	Canadian Health Promotion Survey, 1990 (Statistics Canada)
Height, by sex, among men and women aged 40 years	Mean self-reported height, in kg	Canadian Health Promotion Survey, 1990 (Statistics Canada)

APPENDIX 3. PROCEDURES FOR SELECTING COMPARISON COUNTRIES

A valid comparison country or countries would ideally meet several criteria:

a) Match on key parameters, including being structurally similar to the treated country (i.e., similar initial conditions);
b) Not have received the U.S. Free Trade Agreement 'treatment' in the study period;
c) Exhibit parallel trends in the outcome variable in the period preceding the treatment, and, of course;
d) Have available data.[2]

Figure 1 summarizes how we applied these criteria to identify our sample of comparison countries.

Appendix Figure 3.1. Sample selection procedures.

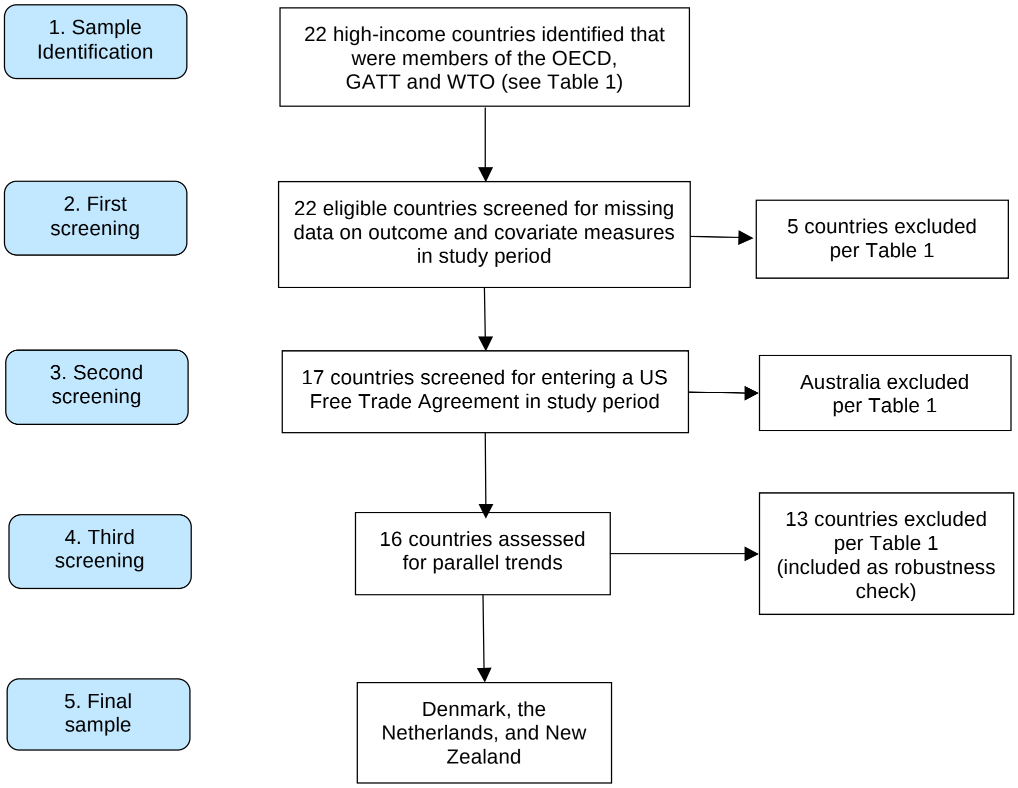

First, we identified 22 potential comparison countries that, like Canada, were classified by the World Bank as "high-income countries" in the study period and were members of the Organization for Economic Co-Operation and Development, an inter-governmental organization designed to stimulate economic progress and world trade. The 22 countries were also members of the General Agreement on Tariffs and Trade (GATT) at the beginning of the study period in 1978 and, from 1995, the World Trade Organization (WTO). The GATT was the WTO's precursor and was a legal agreement between members with an overall purpose of promoting international trade by reducing or eliminating trade barriers such as tariffs or quotas. Thus, these restrictions to the sample of potential comparison countries ensured that countries were structurally similar to Canada in both levels of economic development and openness to trade.

Second, we screened countries for data availability, excluding countries with incomplete calorie availability or covariate data in the study period, 1978–2006. Third, we screened countries for whether they had entered a trade agreement with the U.S. in the study period. Fourth, we screened countries for whether trends in calorie availability were parallel with trends in Canada in the pre-CUSFTA period 1978–1988. This restriction is important as it strengthens the credibility of an assumption implicit in inferences from our fixed-effects models: that calorie availability in Canada would have followed similar trends to those observed in comparison countries had Canada not entered CUSFTA.

Here, we judged calorie availability trends as being parallel to Canada if, like Canada, calorie availability (1) fluctuated slightly in the period 1978–1983 with no overall growth or decline, (2) grew by within 20% of the 200 kcal/ capita/ day rise observed in Canada, 1984–1986, and (3) declined by within 20% of the 90 kcal/ capita/ day decline observed in Canada just before CUSFTA, 1987–1988. In the fifth stage countries meeting all three criteria were included in the analytical sample: Denmark, the Netherlands, and New Zealand.

A table listing the countries we identified as potential comparisons and the reason for each country's exclusion are listed below in Appendix Table 3.1. Appendix 8 summarizes the results from additional analyses for the robustness of our results across alternative comparison country specifications. This includes a sensitivity analysis in which we included countries whose calorie availability trends were just above or below the thresholds for parallel trends, or which met two out of the three parallel trends criteria (column 3 in Appendix 8). The countries included in the additional analysis are denoted with an asterisk in Appendix Table 3.1.

Appendix Table 3.1. Potential Comparison Countries Reasons For Exclusion

Country	Included?	Exclusion criteria
Denmark	No	
Netherlands	No	
New Zealand	No	
Austria	Yes	Not parallel trends
Finland	Yes	Not parallel trends
France	Yes	Not parallel trends
Germany	Yes	Not parallel trends
Greece	Yes	Not parallel trends
Italy	Yes	Not parallel trends
Japan*	Yes	Not parallel trends
Norway*	Yes	Not parallel trends
Portugal	Yes	Not parallel trends
Spain*	Yes	Not parallel trends
Sweden	Yes	Not parallel trends
UK*	Yes	Not parallel trends
U.S.*	Yes	Not parallel trends
Australia	Yes	U.S. FTA
Switzerland	Yes	Missing data
Luxembourg	Yes	Missing data
Ireland	Yes	Missing data
Iceland	Yes	Missing data
Belgium	Yes	Missing data

UK, United Kingdom; FTA, Free Trade Agreement

APPENDIX 4. MODELING WEIGHT GAIN FROM CHANGES TO CALORIC INTAKE

The models developed by Hall and colleagues comprise a series of partial differential equations for estimating weight gain in kilograms from a specified increase in caloric intake, given an individual's height, initial body weight, and physical activity.[3] These dynamic models estimate weight-gain in steady state following a change in caloric intake. This accounts for a variety of physiological adaptations that occur following initial weight gain, such as increases in the expenditure of energy due to the energetic costs of maintaining the newly created tissue. The models also account for metabolic differences according to body fat composition and its variation by age and sex. Separate calculations are required for men and women because women have a higher amount of body fat than do men of similar weight. Thus, whereas sex is not an explicit variable in the equations, specification of the initial body composition at a given body weight differs between men and women. See Hall and Jordan (2008) for a full description of these models.

We applied the models developed by Hall and Jordan to estimate weight gain using a freely available Excel package. The package can be downloaded from the Supplemental Appendix of Hall and Jordan (2008). We estimate the weight gain attributed to a rise in caloric intake of 170 kcal/ capita/ day (assuming 100% pass through from calorie availability to intake) and 85 kcal/ capita/ day (assuming 50% pass through from availability to intake). We estimate this weight gain among 40-year old males and females by entering these calorie intake levels into the spreadsheet together with mean height and body weight data among males and females aged 40 years per the Canadian Health Promotion Survey, 1990. We then calculate weight gain across two physical activity scenarios: low physical activity levels, equivalent to walking 2.2 miles a day at 3–4 miles per hour (mph); and high physical activity, equivalent to walking 17 miles a day at 3–4 mph.

Appendix 5. U.S. investment in the Canadian food and beverage sector, 1982–2006.

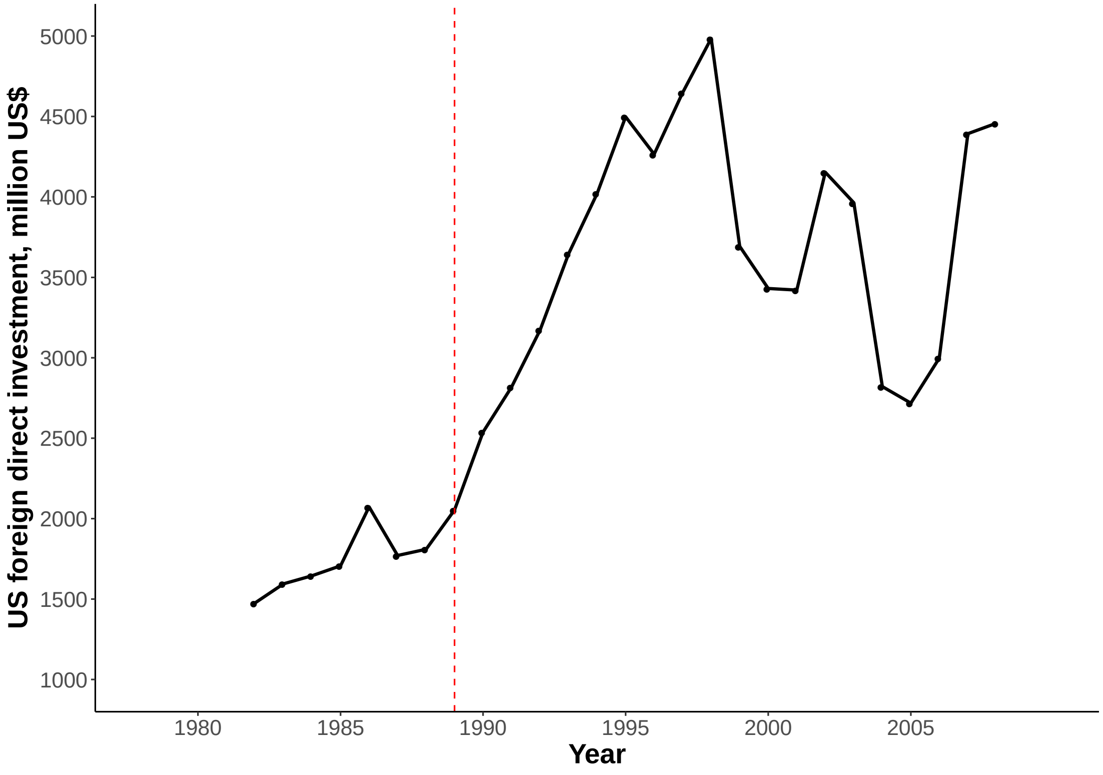

APPENDIX 6. 5-YEAR TIME DELAY BETWEEN CHANGES TO CALORIE AVAILABILITY AND U.S. INVESTMENT IN THE CANADIAN FOOD AND BEVERAGE SECTOR

Trends in calorie availability in Canada did not diverge from comparison countries immediately after CUSFTA. The delay is likely explained by the delayed response of calorie availability to changes in U.S. investment in the Canadian food and beverage sector. Such investments are likely to take time to translate into increased production, sales, and calorie availability due to the time needed to establish or expand production facilities, co-ordinate new distribution networks, increase production and the supply of inputs, and build new sales outlets.[4] Calorie availability in Canada began rising 5 years after the rise in FDI began immediately after CUSFTA, and stopped rising 5 years after the rise FDI stopped (Appendix Figure 6.1). These patterns suggest that changes in calorie availability responded to changes in U.S. investment in the Canadian food and beverage sector at a 5 year time delay.

Appendix Figure 6.1. Trends in calorie availability and U.S. investment in the Canadian food and beverage sector.

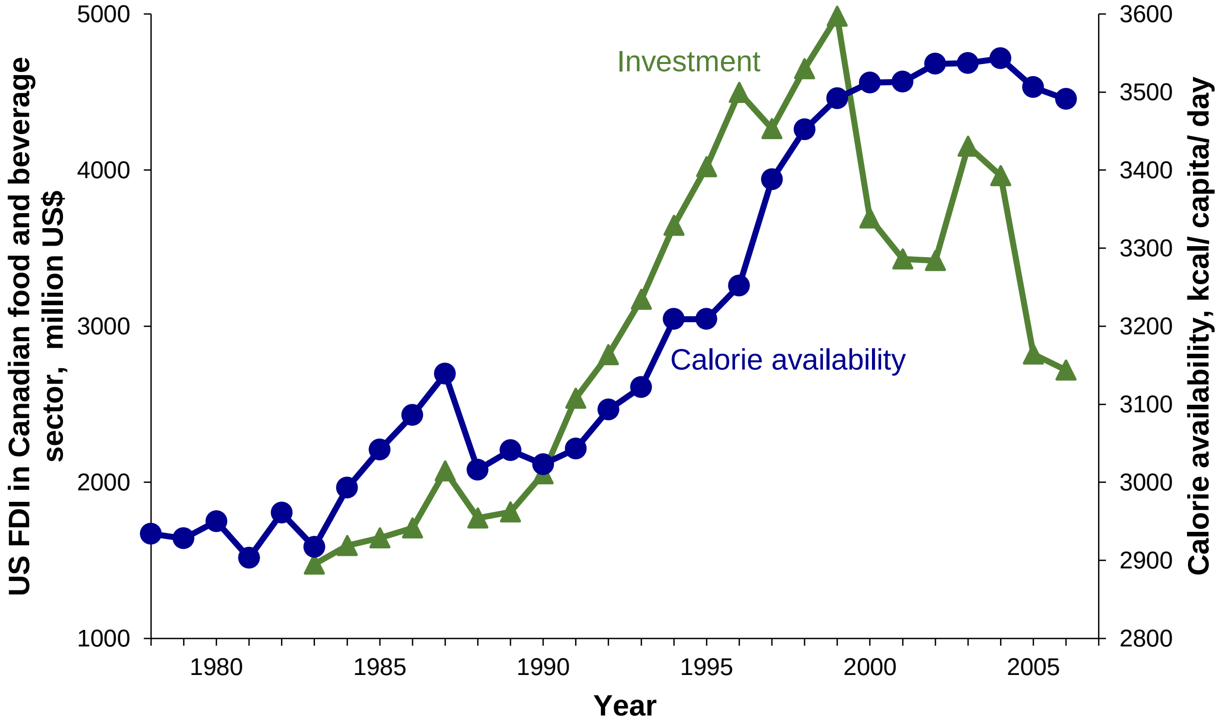

Appendix 7. U.S. imports and exports to the Canadian food and beverage, 1978–2006.

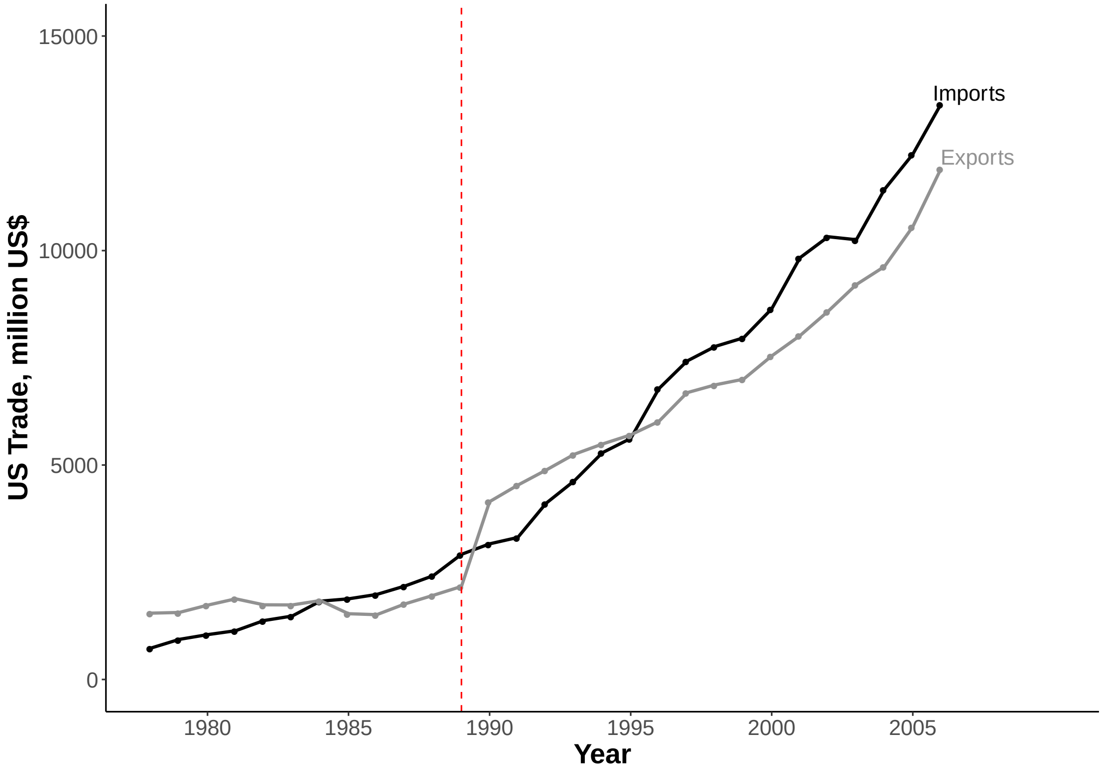

Appendix 8. Estimated Effect of CUSFTA on Calorie Availability in Canada With Additional Comparison Countries: Fixed-Effects Regression Results

**Variable**	**Original sample**	**Original sample plus U.S.**	**Original sample plus near-parallel trends**	**All OECD countries**
CUSFTA, Coefficient (95% CI)	**170.3 (73.0, 267.5)**	**166.2 (74.8, 257.6)**	**211.7 (33.5, 397.9)**	**256.9 (143.3, 370.5)**
US$100 increase in GDP per capita, Coefficient (95% CI)	1.1 (–0.03, 2.3)	2.2 (–1.12, 5.6)	0.6 (–0.7, 2.1)	0.3 (–1.1, 1.7)
1% increase in rate of urbanization, Coefficient (95% CI)	17.1 (–6.5, 40.8)	28.4 (–47.1, 103.8)	–9.9 (–43.3, 23.4)	–6.8 (–36.8, 23.15)
Controls for fixed effects?	**Yes**	**Yes**	**Yes**	**Yes**
Controls for time trends?	**Yes**	**Yes**	**Yes**	**Yes**
Country-years	116	145	261	493
Adjusted R^2	0.62	0.44	0.48	0.61

Notes: Boldface indicates statistical significance (p<0.05). Models were estimated using cluster-robust SEs grouped at the country level.

CUSFTA, Canada U.S. Free Trade Agreement; GDP, Gross Domestic Product; OECD, Organization for Economic Co-Operation and Development.

APPENDIX 9. SYNTHETIC CONTROL METHODOLOGY AND RESULTS

Appropriate specification of a counterfactual is essential for valid causal inference.[5] Inferences based on fixed-effects regressions may be biased as they make comparisons with a combination of countries with characteristics may not resemble Canada before CUSFTA.[6] In addition, inferences based on fixed-effects regressions can be highly sensitive to the choice of model and comparison units and rest on the tenability of the parallel trends assumption. To address these issues and test whether our results are stable in an alternative model with different identifying assumptions that seeks to minimize differences in characteristics between a treated and un-treated unit we re-estimate the impact of CUSFTA using the synthetic method.[7] This method estimates a counterfactual from comparison units using a transparent and data-driven algorithm. The algorithm is designed to select a comparison unit which resembles Canada as closely as possible in the pre-CUSFTA period.

Estimation

Details have been described elsewhere.[7,8] Briefly, we estimate the effect of CUSFTA by comparing calorie availability in Canada with a synthetic control. This synthetic control is estimated using the values of predictors of the outcome – in our case calorie availability – in un-treated comparison countries. The matching algorithm selects comparison units by selecting the combination of countries and weights that minimizes the difference between the value of predictors in the combined synthetic control unit and their observed values in the treated country before the intervention. This minimization algorithm also prioritizes matching on variables that are identified as having the greatest predictive power on the outcome. The estimated effect of CUSFTA is then calculating by comparing the outcomes in the treated unit and the estimated outcomes in the synthetic control in the post-CUSFTA period.

Inference

One limitation of the synthetic control approach is that standard inference testing techniques are not suitable for assessing the significance of the results. Following suggestions by Abadie and colleagues we use placebo tests to compute *p*-values.[8] This approach assigns a dummy treatment to all other countries in the sample that do not actually enter CUSFTA. P-values are then calculated as the proportion of effect estimates that are at least as large as the one obtained for the treated unit. These effect sizes are adjusted for differences in model fit between countries by taking the ratio of the post-CUSFTA effect to the pre-CUSFTA model fit, measured using the Root Mean Squared Prediction Error. There are no rules-of-thumb for evaluating significance levels.[9]

Model and Sample Specification

The variables used for matching countries with Canada to estimate the synthetic control are meant to be predictors of the outcome variable, in our case daily caloric intake, rather than confounders as in the fixed-effects regression. We used data from the World Bank World Development Indicators 2015 Edition[10] and we selected predictors based on existing studies of dietary change and food consumption. These were GDP per capita, GDP growth, and inflation, which can impact consumption levels through their impact on incomes, purchasing power, and preferences and opportunities for consuming calorie dense foods.[11–13] Urbanization is also included as dense networks of consumers provide economies of scale for producers, including fast-food outlets and restaurants that serve calorie-dense meals.[13–15] Since energy needs and dietary preferences vary by age we also included the population in each age group 0–14, 15–64,

and ≥65 years.[16,17] Following recommendations in previous synthetic control analyses, we also capture unobserved heterogeneity between countries by including lagged values of the outcome at fixed at 5 year intervals before CUFSTA was implemented (i.e., in 1978, 1983, and 1988).[7]

We estimate the synthetic control model using two samples: our main sample used for our fixed-effects regression and an expanded sample comprising all structurally similar high-income countries with available data. We expand the sample as, unlike our fixed-effects regressions, we are no longer restricted by the parallel trends assumption. In addition, expanding the sample allows for comparisons across a greater number of units, improving the model fit.[18] We nevertheless keep the sample restricted countries that are structurally similar to Canada before CUSFTA.[7] Thus, the countries in our expanded samples 14 other countries that were also members of the World Trade Organization (WTO) and Organization for Economic Co-operation and Development (OECD), but did not enter into a U.S. FTA during the study period. These countries were: Denmark, Finland, France, Germany, Greece, Italy, Japan, The Netherlands, New Zealand, Norway, Portugal, Spain, Sweden, and the United Kingdom.

Results

Appendix Table 9.1 shows the predictor weights and pre-CUSFTA predictor means in Canada, their mean in our fixed-effects regression sample, and in both estimates of synthetic Canada. The table shows that the synthetic control yields substantial improvements in predictor similarity, especially in terms of the variables with the greatest predictive power.

Appendix Table 9.1. Predictor Weights and Pre-CUSFTA Predictor Means in Canada and Comparison Units

Predictor	Weight	Canada	Main sample	Synthetic Canada, main sample	Synthetic Canada, OECD sample
Calories in 1983	0.38	2,917.0	3,062.50	2,972.0	2,943.3
Calories in 1988	0.28	3,016.0	3,177.00	3,120.0	3,074.4
Population aged >65 years	0.18	9.9	12.99	10.1	10.4
Urbanization	0.07	37.4	30.84	25.0	35.0
Population aged 15–64 years	0.06	68.1	65.29	64.2	66.5
Calories in 1978	0.04	2,934.0	3,113.50	3,124.0	2,972.5
GDP growth	0.00	2.0	1.96	1.6	2.4
GDP per capita	0.00	25,222.6	29,644.42	19,925.7	22,559.6
Inflation	0.00	6.5	7.28	12.4	6.2
Population aged <14 years	0.00	22.0	21.73	25.7	23.1

CUSFTA, Canada U.S. Free Trade Agreement; GDP, Gross Domestic Product; OECD, Organization for Economic Co-Operation and Development.

Appendix Table 9.2 shows the estimated impact of CUSFTA from the synthetic control models, the estimated *p*-values of these estimates, and the model fit in the pre-CUSFTA period. Table 2 shows that in both samples the effect estimate of CUSFTA exceeded placebo effects in all un-treated countries, bolstering our confidence that the observed effect is driven by CUSFTA.

Appendix Table 9.2. Impact of CUSFTA on Calorie Availability in Canada: Synthetic Control Results

Variable	Fixed-effects sample	Full OECD sample
CUSFTA effect	171.5	249.1
p-value[a]	1/4 = 0.25	1/15 = 0.07
Pre-CUSFTA RMSPE[b]	107.9	30.9

[a]Note that the *p*-values presented here do not have the standard interpretation; Appendix 2 provides a full description of the synthetic control methodology including methods for estimating *p*-values and their interpretation.
[b]RMSPE = Root Mean Squared Predictor Error, a measure of the model fit. In the main sample synthetic Canada is estimated entirely from data in New Zealand. In the OECD sample synthetic Canada is estimated from a weighted combination of Japan (33%), New Zealand (32%), the Netherlands (20%), and Portugal (15%).

CUSFTA, Canada U.S. Free Trade Agreement; OECD, Organization for Economic Co-Operation and Development.

Appendix 10. In-time Placebo Analysis and Test for Impact of Pre-Intervention Dip

**Variable**	**In-time placebo**	**Pre-intervention dip**
CUSFTA, Coefficient (95% CI)	–5.0 (–92.1, 82.0)	248.1*** (137.2, 358.9)
US$100 increase in GDP per capita, Coefficient (95% CI)	1.6 (–0.3, 3.6)	1.13 (–0.01, 2.2)
1% increase in rate of urbanization, Coefficient (95% CI)	16.5 (–17.5, 50.4)	6.4 (–18.0, 30.9)
Controls for fixed effects?	Yes	Yes
Controls for time trends?	Yes	Yes
Country-years	44	100
Adjusted R^2	0.35	0.67

Notes: Boldface indicates statistical significance (**p*<0.1; ***p*<0.05; ****p*<0.001).

CUSFTA, Canada U.S. Free Trade Agreement; GDP, Gross Domestic Product

Appendix 11. Leave-one-out analysis

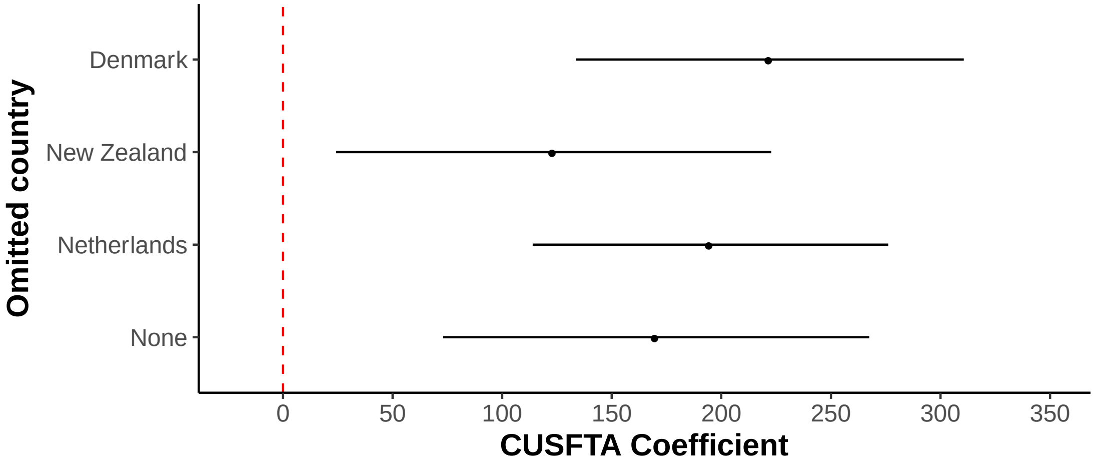

APPENDIX REFERENCES

1. Government of Canada. The Canada-U.S. Free Trade Agreement. Canada; 1989.
2. Angrist JD, Pischke J. *Mostly Harmless Econometrics: An Empiricist's Companion*. Princeton University Press, 2009.
3. Hall KD, Jordan PN. Modeling weight-loss maintenance to help prevent body weight regain. *Am J Clin Nutr*. 2008;88(6):1495–1503.
4. Krugman PR. *International economics: Theory and policy*. 8th ed. Pearson Education India, 2008.
5. King G, Keohane RO, Verba S. *Designing Social Inquiry: Scientific Inference in Qualitative Research*. Princeton University Press, 2001:29.
6. King G, Zeng L. The dangers of extreme counterfactuals. *Polit Anal*. 2006;14(2):131–159. https://doi.org/10.1093/pan/mpj004.
7. Abadie A, Gardeazabal J. The economic costs of conflict: A case study of the Basque country. *Am Econ Rev*. 2003;93(1):113–132. https://doi.org/10.1257/000282803321455188.
8. Abadie A, Diamond A, Hainmueller J. Comparative politics and the synthetic control method. *Am J Pol Sci*. 2015;59(2):495–510. https://doi.org/10.1111/ajps.12116.
9. Ho DE, Imai K. Randomization inference with natural experiments. *J Am Stat Assoc*. 2006;101(475):888–900. https://doi.org/10.1198/016214505000001258.
10. World Bank. World Development Indicators 2015. World Bank. Published 2015.
11. Drewnowski A, Popkin BM. The nutrition transition: new trends in the global diet. *Nutr Rev*. 1997;55(2):31–43. https://doi.org/10.1111/j.1753-4887.1997.tb01593.x.
12. Drewnowski A, Darmon N. The economics of obesity: dietary energy density and energy cost. *Am J Clin Nutr*. 2005;82(1 suppl):265S–273S.
13. Stuckler D. Population causes and consequences of leading chronic diseases: A comparative analysis of prevailing explanations. *Milbank Q*. 2008;86(2):273–326. https://doi.org/10.1111/j.1468-0009.2008.00522.x.
14. Popkin BM. Urbanization, lifestyle changes and the nutrition transition. *World Dev*. 1999;27(11):1905–1916. https://doi.org/10.1016/S0305-750X(99)00094-7.
15. Schram A, Labonte R, Sanders D. Urbanization and international trade and investment policies as determinants of noncommunicable diseases in sub-Saharan Africa. *Prog Cardiovasc Dis*. 2013;56(3):281–301. https://doi.org/10.1016/j.pcad.2013.09.016.
16. Cooke LJ, Wardle J. Age and gender differences in children's food preferences. *Br J Nutr*. 2007;93(5):741. https://doi.org/10.1079/BJN20051389.
17. Wansink B, Cheney MM, Chan N. Exploring comfort food preferences across age and gender. *Physiol Behav*. 2003;79(4–5):739–747. https://doi.org/10.1016/S0031-9384(03)00203-8.
18. Billmeier A, Nannicini T. Assessing economic liberalization episodes: a synthetic control approach. *Rev Econ Stat*. 2013;95(3):983–1001. https://doi.org/10.1162/REST_a_00324.
